# Effects of hesperidin and ascorbic acid combination on boar sperm quality during cryopreservation

**DOI:** 10.3389/fvets.2025.1573983

**Published:** 2025-07-04

**Authors:** Ruilan Dong, Yupeng Dou, Min Zhang, Lan Luo, Guanghui Yu

**Affiliations:** ^1^College of Animal Science and Technology, Qingdao Agricultural University, Qingdao, China; ^2^General Station of Animal Husbandry of Shandong Province, Jinan, China

**Keywords:** antioxidant, ascorbic acid, boar sperm cryopreservation, hesperidin, sperm quality

## Abstract

Hesperidin or ascorbic acid is an effective antioxidant that can protect frozen-thawed sperm from oxidative damage. The aim of the present study was to investigate the cryoprotective effects of hesperidin (Hsd) and ascorbic acid (AsA) on boar sperm quality during cryopreservation. Sperm samples were collected once a week for 3 weeks. The ejaculated semen from eight boars in each of three replicates were pooled and split into seven groups with different doses of Hsd (0, 15, 45) μM and/or AsA (0, 100, 300) μM added to the freezing extender. The sperm motility, membrane integrity, acrosome integrity, mitochondrial membrane potentials, DNA integrity, reactive oxygen species (ROS), malondialdehyde (MDA) concentration, antioxidant enzyme activities, and protein expression were measured after freezing-thawing to evaluate the qualitative parameters of boar sperm. The results showed that the sperm motility, plasma membrane integrity, acrosomal integrity, DNA integrity, and mitochondrial membrane potential were significantly improved in the 15 μM Hsd + 100 μM AsA group compared with the control and other treatment groups (*p* < 0.01). It was observed that the supplementation in the 15 μM Hsd + 100 μM AsA group significantly improved the ATP content and antioxidant enzyme activities (SOD, CAT, GSH-Px, and PRODH) (*p* < 0.01). The levels of MDA and ROS were significantly reduced in the 15 μM Hsd + 100 μM AsA group (*p* < 0.01). Moreover, the supplementation of Hsd combined with AsA significantly increased the expression of the anti-apoptosis protein Bcl-2 and decreased the expression of the pro-apoptotic protein caspase-3, P53, Bax, and Cytochrome C (*p* < 0.05). These findings demonstrated that supplementing the freezing extender with both Hsd and AsA had a combined, beneficial effect on the quality of frozen-thawed boar sperm. The combination of 15 μM Hsd + 100 μM AsA showed greater potential in protecting the boar sperm during cryopreservation than the separate addition of either Hsd or AsA. The supplementation of Hsd and AsA could improve the quality of frozen-thawed sperm as an antioxidant.

## 1 Introduction

Cryopreservation of boar sperm is a reliable choice for long-term preservation of important genetic materials, and it is a convenient way to utilize outstanding individuals in the country and abroad (1, 2). However, a number of parameters, including diluents, the rate of freezing and thawing, cryoprotectants, breeds and individual boars, can easily alter the survival of frozen-thawed boar sperm (3). The efficiency of artificial insemination with frozen-thawed sperm is low, accounting for only 1% of swine insemination due to its poor efficiency (4). The polyunsaturated fatty acid (PUFA) content in the boar sperm membrane is particularly rich (5). This renders the boar sperm highly susceptible to lipid peroxidation (LPO), which occurs as a result of the oxidation of membrane lipids by partially reduced oxygen molecules, such as superoxide, hydrogen peroxide, and hydroxyl radicals (6). LPO influences the integrity and other properties of the sperm plasma membrane, even giving rise to the formation of malondialdehyde (MDA), the lipid peroxidation product of unsaturated fatty acid. During cryopreservation, freezing stress can disrupt the membrane of boar sperm and increase the production of reactive oxygen species (ROS). The release of ROS after thawing further aggravates oxidative stress. Oxidative stress has numerous detrimental effects on boar sperm, leading to DNA damage and impairing the production of ATP, which is essential for sperm motility. It also triggers the apoptosis pathway through ROS, leading to a decrease in the antioxidant capacity of boar sperm, making them more vulnerable to oxidative damage, finally depriving sperm of motility and viability (7). The quality of frozen-thawed sperm (e.g., sperm motility, membrane integrity, acrosome integrity, and mitochondria activity) may be influenced by a number of variables, such as species, ejaculates, diluent composition, cryopreservation techniques, and cryoprotective agents (8). Therefore, it is of great importance and urgency to select effective and nontoxic antioxidants to improve the boar sperm quality during cryopreservation. The addition of antioxidants such as hesperidin and ascorbic acid to freezing extenders has a positive effect on the cryopreservation quality of frozen sperm (9).

Flavonoids are the largest family of phenolic compounds, the majority of which are hesperidin (Hsd) (10). Hsd is widely distributed in various citrus fruits and plays an important role in many biological and physiological activities (11). Hsd may play an additive or synergistic role in anti-oxidation, anti-inflammatory, anticancer, and cardiovascular protection activities (12, 13). Hsd had greater antioxidative effects on fresh and frozen-thawed semen quality in Simmental bull (14). Ascorbic acid (AsA) is the most effective and least toxic antioxidant, involved in vital biological activities (15). It is an antioxidant that has showed positive effects during the vitrification of porcine embryos (16). Previous studies have shown that the supplementation of AsA (17) or AsA-derivative (2-O-α-glucoside, AA-2G) (18) improves sperm motility and survival and reduces MDA and DNA damage in boar.

The aim of this study was to determine the positive effects of the addition of Hsd and AsA (i.e., alone or combined) on boar frozen-thawed sperm quality, including sperm motility, membrane integrity, acrosome integrity, mitochondrial membrane potentials, DNA integrity, ROS level, MDA concentration, antioxidant enzyme activities [superoxide dismutase (SOD), catalase (CAT), glutathione peroxidase (GSH-Px), and proline dehydrogenase (PRODH)], and apoptotic factor protein expression.

## 2 Materials and methods

### 2.1 Animal and sperm collection

The ejaculate sperm samples used in this study were collected from eight healthy Duroc boars (aged 1.5 to 2.5 years) from a boar station. The boars were raised in separate limit bar (3 m long × 2.5 m wide) and fed 2.5 to 3.0 kg of feed twice a day with ad libitum water. The sperm was collected at 8:00 am after feeding for 1 h using the “gloved-hand” method once a week for three successive weeks. The gel particle was removed with four layers of sterile gauze, and only the sperm-rich fractions were brought to the laboratory within 60 min at 17°C. The sperm samples were assessed microscopically, and more than 90% of motile sperm and less than 10% of abnormal sperm were used in the trial. The ejaculated semen from eight boars was pooled to eliminate individual differences and divided into seven groups with three replicates per group.

### 2.2 Chemicals and extenders

Unless otherwise specified, all chemicals and reagents were purchased from Sinopharm Group in China. The Modena solution was prepared in the laboratory containing 153 mM D-glucose, 26.7 mM trisodium citrate, 11.9 mM sodium hydrogen carbonate, 15.1 mM citric acid, 6.3 mM ethylene diamine tetraacetic acid (EDTA)-2 Na, 46.6 mM Tris, 1,000 IU/mL penicillin G sodium salt (Solarbio Institute of Biotechnology, China), and 1 mg/mL streptomycin sesquisulfate (Solarbio Institute of Biotechnology, China), as described by Dong et al. (19). Extender I was composed of 11% lactose solution and egg yolk (4:1, v:v), containing seven groups of different concentrations of Hsd (97% purity; Sigma-Aldrich Co., St. Louis, Missouri, United States) and/or AsA (Cat: A8100, 99% purity; Solarbio Institute of Biotechnology, China). The concentrations were referred to Tahmasbian et al. (14) and Giaretta et al. (17) (Table 1). Extender II was composed of extender I (containing different concentrations of Hsd and/or AsA), glycerol, and Orvus Ex Paste^™^ (Minitube, Germany) (95.5%:3%:1.5%, v:v:v).

**Table 1:** Experimental design of Hsd and AsA concentrations in different groups.

Groups	Control	H45	A300	H15 + A300	H15 + A100	H45 + A300	H45 + A100
Hsd (μM)	0	45	0	15	15	45	45
AsA (μM)	0	0	300	300	100	300	100

Hsd, hesperidin; AsA, ascorbic acid. H15 and H45 refer to the addition of 15 and 45 μM of hesperidin, respectively; A100 and A300 refer to the addition of 100 and 300 μM of ascorbic acid, respectively.

### 2.3 Sperm processing

According to Dong et al. (19), the fresh semen was diluted with Modena solution (v:v = 1:1) and incubated for 2 h at 17°C. It was centrifuged at 900 × g for 10 min to remove the Modena solution. The sperm pellets were resuspended with extender I that contain different concentrations of Hsd and AsA at a concentration of 2 × 10^9^ sperm/mL and cooled slowly from 17 to 5°C for 2 h. Subsequently, the sperm was resuspended with the same volume of freezing extender II and packed into 0.5 mL plastic straws. The sealed straws were placed horizontally on a rack and frozen for 10 min in a vapour 3 cm above the liquid nitrogen, and then the straws were plunged into the liquid nitrogen for storage. After 2 weeks of storage, the extended semen was thawed in a water bath at 50°C for 10 s and then resuspended in Modena (1:4, v:v, 37°C) to evaluate the quality of sperm at 10 min after incubation at 37°C. Sperm samples were used to determine the qualitative parameters of boar sperm in the following analysis.

### 2.4 Assessments of sperm motility after freezing-thawing

Sperm motility was evaluated using the computer-assisted sperm analysis (CASA) system (QH-III, Spain). The associated parameters included the total motile sperm (TM, %), motile progressive sperm (PM, %), curvilinear velocity (VCL, μm/s), straight-line velocity (VSL, μm/s), average path velocity (VAP, μm/s), and linearity of the curvilinear trajectory (LIN, ratio of VSL/VCL, %), which were all evaluated. According to Dong et al. (19), 5 μL semen samples were placed on an analyzer’s Makler chamber and maintained at 37°C for 10 min during the analysis. Sperm motility was estimated by calculating the percentage of sperm showing progressive movement after viewing five different fields, examining over 500 sperm.

### 2.5 Measurement of sperm membrane integrity, acrosome integrity, and DNA integrity after freezing-thawing

**Figure 1:**
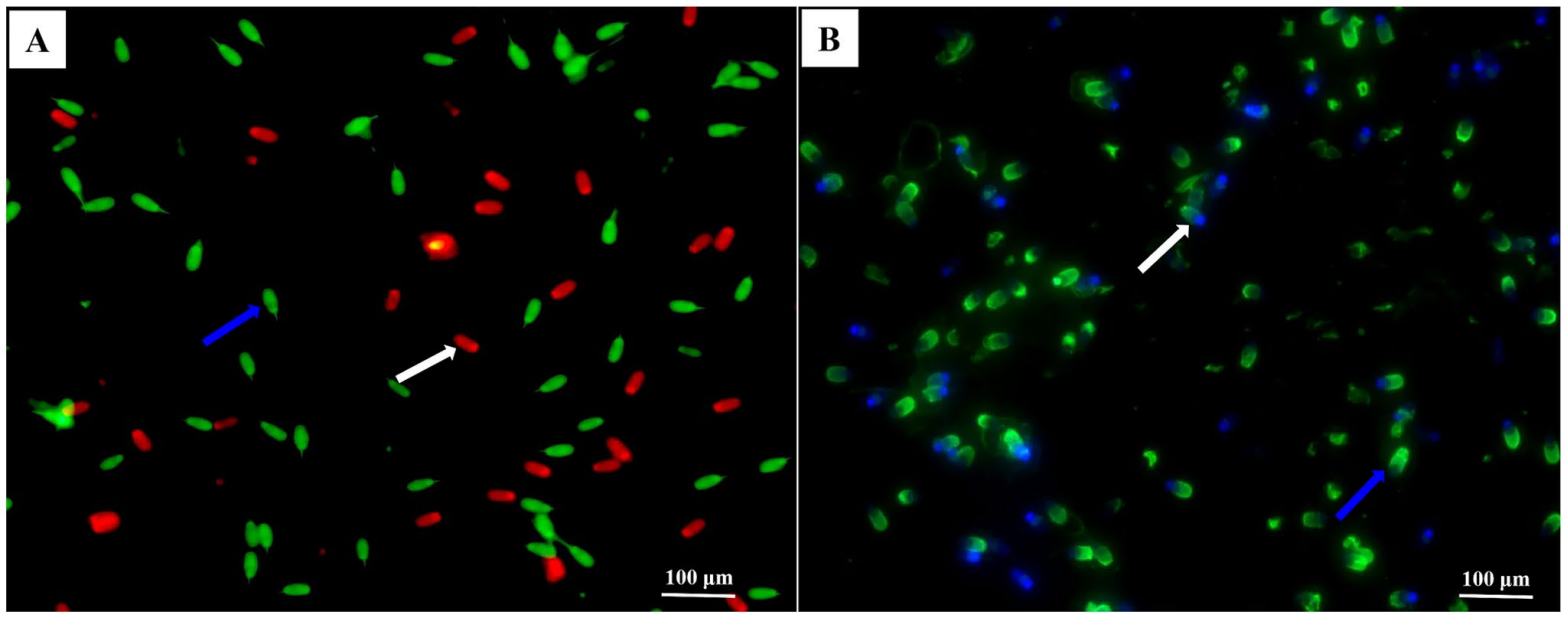
Photomicrographs of the post-thawed sperm stained with membrane using the SYBR-14/PI and acrosomes fluorescein isothiocyanate-peanut agglutinin (FITC-PNA). Images **(A,B)** obtained under a phase-contrast microscope. In image **(A)**, the blue arrow indicated membrane integrity, and the white arrow indicated damaged membrane. In image **(B)**, the blue arrow indicated intact acrosomes and sperm with intensively bright fluorescence of the acrosomal cap, which indicated by an intact outer acrosomal membrane. The white arrow indicated damaged acrosome and sperm with blue fluorescence, which indicated a loss of the outer acrosomal membrane (bars = 100 μm).

Membrane integrity was determined using the SYBR-14/PI sperm viability kit (L7011, Invitrogen^™^, Thermo Fisher Scientific) according to Zhang et al. (20). For this purpose, an aliquot of 250 μL sample was stained with 25 μL SYBR-14 working solution (100 mM in DMSO) in 36°C darkness for 10 min, followed by the addition of 12.5 μL PI stock solution (2.4 mM) and incubated for another 10 min. Staining was monitored and photographed using an epifluorescence microscope (DMi8, Leica, Germany) with a 400 × fold filter, and a total of 200 sperm in each slide was examined. Sperm were classified into two groups (Figure 1A), i.e., intact membranes and damaged membranes.

Acrosome integrity was evaluated using fluorescein isothiocyanate-peanut agglutinin (FITC-PNA, L7381, Sigma), as described by Zhang et al. (20). To do so, a 300 μL aliquot of each sperm sample was smeared onto a clean glass slide and then fixed with absolute methanol for 10 min. Each slide was then coated with 30 μL FITC-PNA solution (100 μg/mL) diluted with phosphate-buffered saline (PBS). The slides were incubated in a dark and moist chamber at 37°C for 30 min. Subsequently, the slides were rinsed with PBS, air-dried in the dark, and then mounted with 600 μL antifade solution to maintain fluorescence. Samples were monitored and photographed using an epifluorescence microscope (DMi8, Leica, Germany) with a 400 × fold filter, and a total of 200 sperm in each slide was examined. As shown in Figure 1B, fluorescence images of sperm stained with FITC-PNA could be classified into two groups, i.e., intact acrosomes (sperm with intensively bright fluorescence of the acrosomal cap) and damaged acrosomes (sperm without fluorescence).

**Figure 2:**
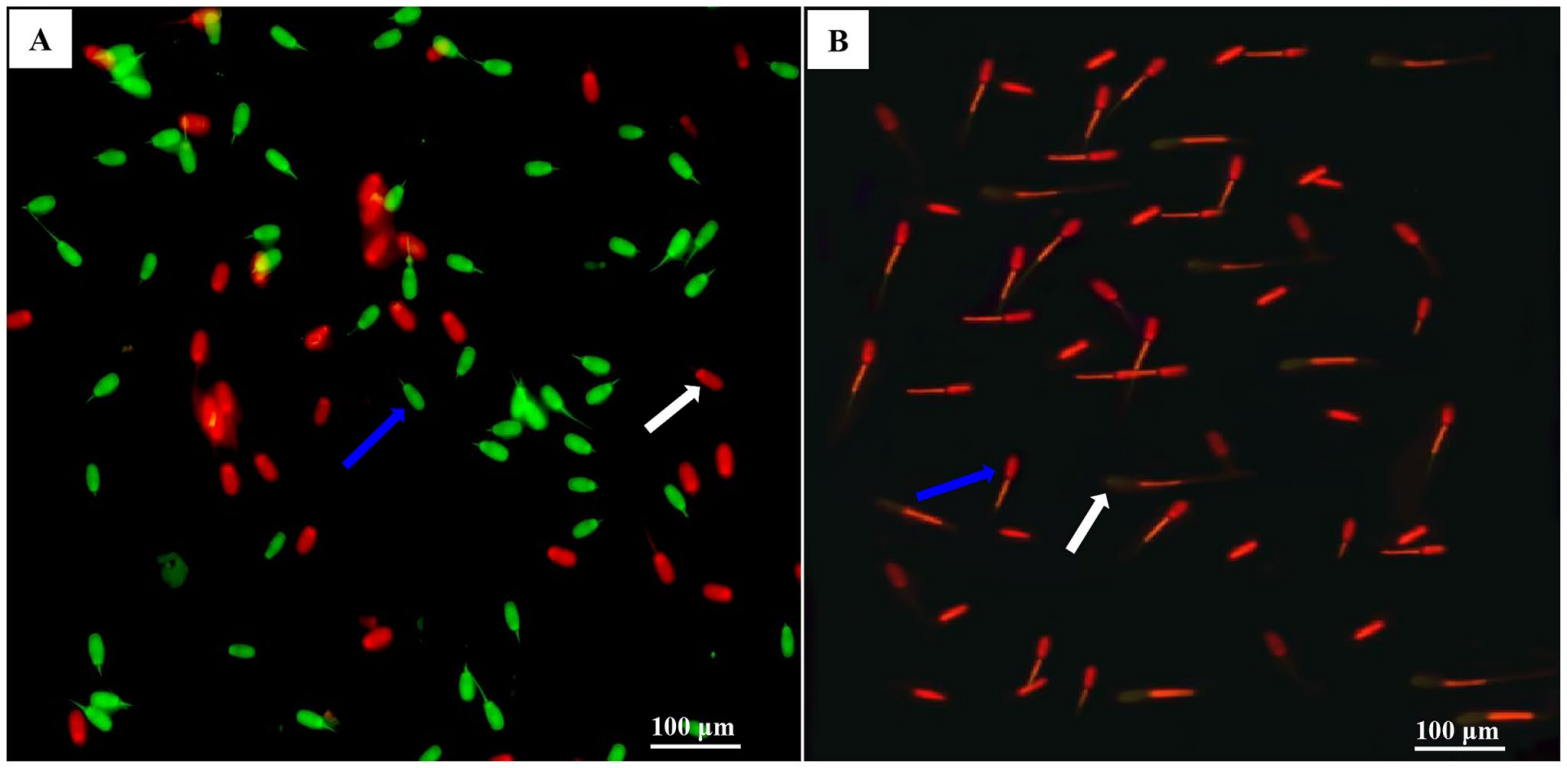
Photomicrographs of the post-thawed sperm stained with MMP (mitochondrial membrane potentials) using JC-1 fluorescent probes and DNA using acridine orange (AO) staining. Images **(A,B)** were obtained under a phase-contrast microscope. In image **(A)**, the blue arrow indicated intact DNA and sperm with intensively bright green fluorescence of the DNA, which indicated by an intact DNA integrity. The white arrow indicated damaged DNA and sperm with red fluorescence, which indicated DNA damage. In image **(B)**, the blue arrow indicated sperm with a high level of MMP (high orange fluorescence level), and the white arrow indicated sperm with a low MMP level (low orange fluorescence level).

DNA integrity was determined using acridine orange (AO) staining (CA1142, Solarbio Institute of Biotechnology, China). According to our previous study (19), an aliquot of a 30 μL sperm sample was washed with AO stain buffer (1×). The sperm concentration was adjusted to 10^6^ sperm/mL by resuspension with an appropriate volume of AO stain buffer (1×). Appropriate amount of sperm cell suspension were mixed with AO staining (19:1) and incubated at 37°C for 15 min. The sperm were spread on each slide and photographed under an epifluorescence microscope (DMi8, Leica, Germany) with a 400× magnification filter. In each slide, 200 sperm were examined, and the percentage of sperm with healthy double-stranded DNA (normal, green fluorescent) or single-stranded DNA (abnormal, red fluorescent) was observed, as shown in Figure 2A.

### 2.6 Assessments of sperm mitochondrial activity after freezing-thawing

Sperm mitochondrial activity was determined using a Mitochondrial Membrane Potential Detection Kit (C2003S, Beyotime Institute of Biotechnology, China), as described by Dong et al. (19). Each sperm sample (2 × 10^6^ sperm/mL) was stained with a JC-1 working solution and incubated in the dark at 37°C for 30 min. They were centrifuged at 600 × g at 4°C for 5 min and then resuspended on ice with JC-1 buffer solution. The stained samples were immediately evaluated under a fluorescence microscope (DMi8, Leica, Germany) with a set of 400 × fold filters, and a total of 200 sperm in each slide was examined. High levels of orange fluorescence emission are associated with the sperm mid-piece (where the mitochondria are located), indicating high mitochondrial activity (Figure 2B). The heads of the sperm emit light yellow, indicating low mitochondrial activity.

### 2.7 Measurement of sperm intracellular ROS, MDA, and ATP content after freezing-thawing

The ROS content was measured using a Reactive Oxygen Species Assay Kit (D6470, Solarbio Institute of Biotechnology, China) according to our previous study (19). Semen samples were suspended in PBS containing 10 μM DCFH-DA, and incubated at 37°C in the dark for 20 min (5 × 10^6^ sperm/mL). Relative fluorescence levels were qualified using a fluorospectro photometer at 488 nm excitation and 525 nm emission (F-4500, Implen, Japan).

The MDA content was measured using a Malondialdehyde Assay Kit (A003-1-2, Nanjing Jiancheng Bioengineering Institute, China) according to the manufacturer’s instructions. Semen samples (10^8^ sperm/group) were lysed in 100 μL of cell lysis reagent. After 10 min centrifugation at 10,000 × g, the supernatant was used to determine the MDA. The absorbance at 532 nm was recorded using a microplate reader (Infinite M Nano, Tecan, Switzerland).

ATP levels of sperm were assessed using an ATP Assay Kit (S0027, Beyotime Institute of Biotechnology, China). According to Dong et al. (19), sperm samples (10^7^ sperm) were lysed with 200 μL of schizolysis solution and ultrasonic cell crusher (20 KHz, 750 W, operating at 40% power, five cycles of 3 s on and 5 s off), and centrifuged at 4°C at 12,000 × g for 10 min. The supernatants were immediately collected, transferred to 96-well plates, and measured using a luminometer (GloMax 20/20, Promega, United States).

### 2.8 Measurement of sperm GSH-Px, SOD, catalase and PRODH activities after freezing-thawing

The Glutathione Peroxidase Assay Kit (A005-1-2), Superoxide Dismutase Assay kit (A001-3-2), Catalase Assay kit (A007-2-1) (Nanjing Jiancheng Institute of Biotechnology), and Proline Dehydrogenase Assay Kit (BC4160, Solarbio Institute of Biotechnology, China) were used to measure the GSH-Px, SOD, CAT, and PRODH activities of sperm, respectively, according to the manufacturer’s instructions. The sperm samples were centrifuged at 1,000 × g at room temperature for 5 min. After being rinsed with PBS for 3 times and re-suspended to 1 mL PBS, it was lysed ultrasonically (20 KHz, 750 W, operating at 40% power, five cycles of 3 s on and 5 s off) on ice and centrifuged at 12,000 × g at 4°C for 10 min. The supernatants were transferred to a 96-well plate to detect the activities of GSH-Px, SOD, CAT, and PRODH with three replicates in each group.

### 2.9 Western blotting

Total protein was extracted from thawed sperm with a concentration of 2 × 10^9^ sperm/mL, with protein lysis buffer composed of 20 mM Tris (pH7.5), 150 mM NaCl, 1% Triton X-100, PMSF [phenylmethanesulfonyl fluoride (long term storage to inhibit protease)] and the concentration was measured using a BCA Protein Assay Kit (P0012S, Beyotime Institute of Biotechnology, China) according to Zhu et al. (21). About 20 μg of total protein in each sample was fractionated using 10% SDS-PAGE. After transferring the resolved protein onto 0.2 μm PVDF membranes, the membrane was blocked with 5% nonfat milk and incubated with anti-α-tubulin (1:1000, Cat No. A6830), anti-B-cell lymphoma protein-2 (1:1000, Cat No. A21873), anti-BCL2-Associated X (1:1000, Cat No. A19648), anti-P53 (1:1000, Cat No. A19585), anti-caspase-3 (1:1000, Cat No. A19654), and anti-Cytochrome C (1:1000, Cat No. A4912) overnight at 4°C. All antibodies were type of polyclonal, host species of rabbit, purchased from ABclonzl, Wuhan, China. The membranes were washed with 1 × TBST 3 times and further incubated with HRP-conjugated secondary antibody (1:1000, type of polyclonal, host species of rabbit, Cat No. AS014) (ABclonzl, Wuhan, China) at room temperature for 1 h. Enhanced chemiluminescence (ECL) detection was performed using the ECL^™^ Prime Western Blotting Detection Reagents (P0018FS, Beyotime Institute of Biotechnology, China) according to the manufacturer’s instruction and appropriate exposure of blots to Fuji X-ray film (iBright FL1000, ABI, United States). Image acquisition and densitometric analysis of gels, blots and membranes were performed using ImageLab software (version 4.1, Bio-Rad).

### 2.10 Statistical analysis

Data were expressed as mean ± standard error of the mean (SEM). The effects of Hsd and/or AsA treatments were analyzed using one-way ANOVA analysis using general linear model (GLM) procedure in SAS (SAS Institute, Inc., Cary, NC). The Tukey’s post-hoc test was performed after a statistically significant omnibus test to compare multiple treatment groups (SAS Institute, Inc., Cary, NC). The level of statistical significance was set at *p* < 0.05.

## 3 Results

### 3.1 Effects of supplementation of Hsd and AsA on frozen-thawed boar sperm motility

Compared with the control group (CON), total and progressive sperm motilities were significantly increased in all treatment groups (*p* < 0.01, Table 2). Sperm motility after thawing with 15 μM Hsd + 100 μM AsA was significantly (*p* < 0.01) higher than that of other treatments. Similarly, the straight-line velocity (VSL) and curvilinear velocity (VCL) of frozen-thawed sperm in the addition of 15 μM Hsd + the 100 μM AsA group were significantly improved compared with control, Hsd, and AsA groups (*p* < 0.01). In addition, the average path velocity (VAP) and linearity of frozen-thawed sperm were significantly increased (*p* < 0.01), except for AsA.

**Table 2:** Effects of different concentrations of hesperidin and ascorbic acid on post-thawed sperm motility parameters in control and at 10 min after thawing.

Items	Treatment							*p*-value
	Control	H45	A300	H15 + A300	H15 + A100	H45 + A300	H45 + A100	
Total motility, %	48 ± 0.9^e^	58 ± 0.3^c^	52.3 ± 0.6^d^	57.2 ± 0.4^c^	67.3 ± 0.6^a^	53.9 ± 0.7^d^	63.0 ± 1.3^b^	<0.01
Progressive motility, %	32.2 ± 0.7^e^	42.1 ± 0.8^c^	36.9 ± 0.2^d^	42.1 ± 0.9^c^	53.7 ± 1.1^a^	41.5 ± 0.6^c^	48.8 ± 0.9^b^	<0.01
VCL, μm/s	90.5 ± 1.0^f^	104.5 ± 1.6^d^	99.3 ± 1.7^e^	105.7 ± 0.9^d^	135.8 ± 0.8^a^	112.1 ± 1.6^c^	125.9 ± 0.7^b^	<0.01
VSL, μm/s	21.7 ± 0.8^d^	35.2 ± 0.3^b^	27.0 ± 1.2^c^	32.8 ± 1.0^b^	44.6 ± 0.9^a^	35.9 ± 1.1^b^	41.7 ± 1.3^a^	<0.01
VAP, μm/s	71.7 ± 1.0 ^e^	81.8 ± 0.4^d^	73.8 ± 2.9^e^	88.7 ± 0.3^c^	105.3 ± 0.5^a^	93.2 ± 0.6^b^	95.1 ± 0.9^b^	<0.01
LIN, %	24.0 ± 1.2^b^	32.8 ± 0.7^a^	27.2 ± 1.6^b^	31.0 ± 0.7^a^	33.7 ± 0.9^a^	32.1 ± 1.3^a^	33.2 ± 0.9^a^	<0.01

Values denote the mean ± standard error (SEM). H15 and H45 refer to the addition of 15 and 45 μM of hesperidin, respectively; A100 and A300 refer to the addition of 100 and 300 μM of ascorbic acid, respectively. Different superscript letters in the same line indicate significant differences (*p* < 0.05). TM, total motility; PM, progressive motility; VCL, curvilinear velocity; VSL, straight-line velocity; VAP, average path velocity; LIN, linearity.

### 3.2 Effects of supplementation with Hsd and AsA on the physiological characteristics and ATP content of frozen-thawed boar sperm

**Figure 3:**
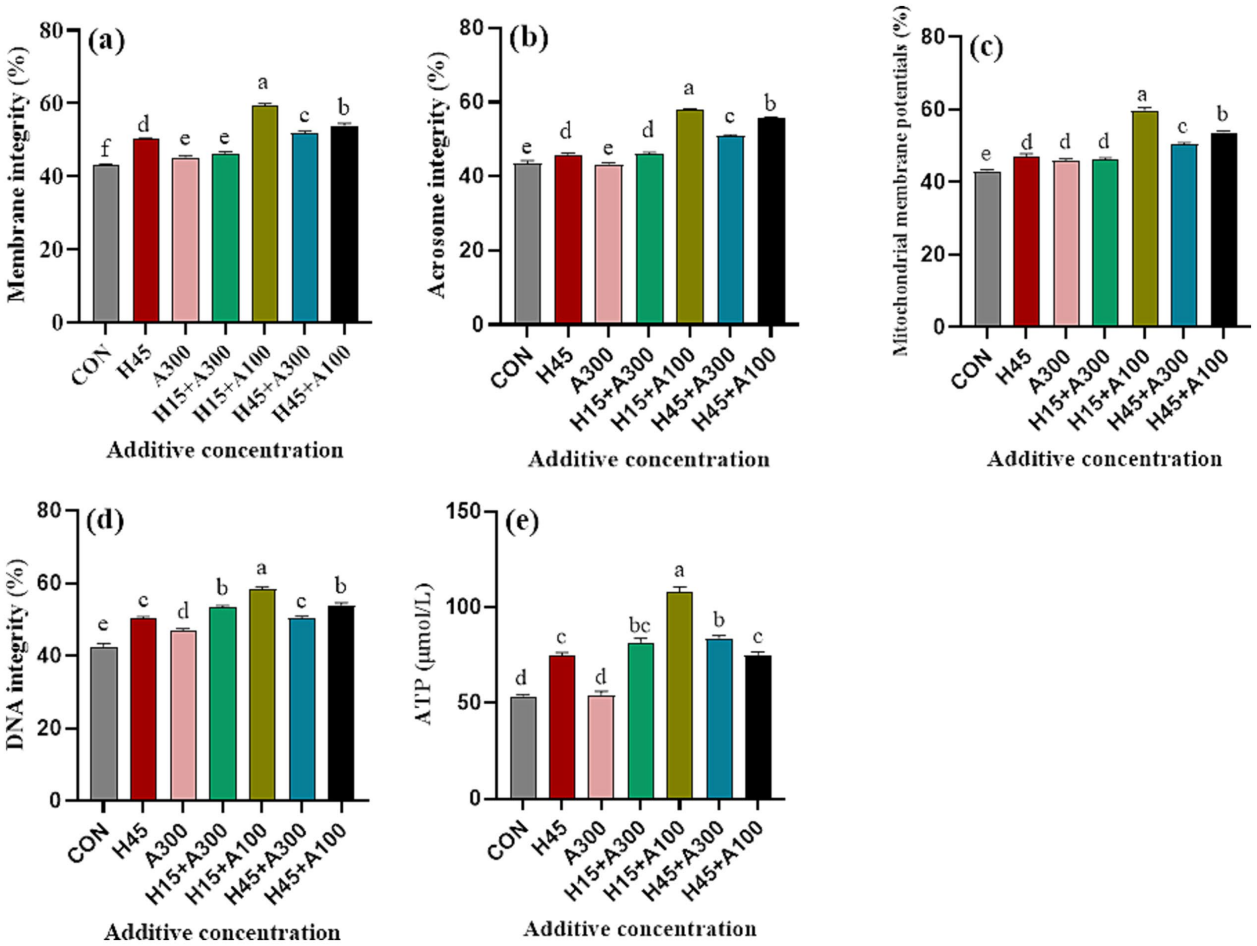
Effects of Hsd and AsA supplementation on the physiological characteristics and ATP content of frozen-thawed boar sperm in control and at 10 min after thawing. **(a)** Membrane integrity, **(b)** acrosome integrity, **(c)** mitochondrial membrane potentials, **(d)** DNA integrity, **(e)** ATP content.

The supplementation of Hsd and AsA to the extender significantly improved the membrane, acrosome, DNA integrity and the mitochondrial membrane potential of frozen-thawed sperm compared with the control group (Figure 3, *p* < 0.001). In addition, ATP levels were significantly (*p* < 0.01) greater in the Hsd-containing treatment compared with the control and groups that only contained AsA (Figure 3). Moreover, ATP content, mitochondrial membrane potential, membrane, acrosomal and DNA integrity of frozen-thawed sperm in the 15 μM Hsd + 100 μM AsA group were significantly (*p* < 0.01) higher than those in the control and other treatment groups (Figure 3).

### 3.3 Effects of supplementation with Hsd and AsA on antioxidant parameters of frozen-thawed boar sperm

**Figure 4:**
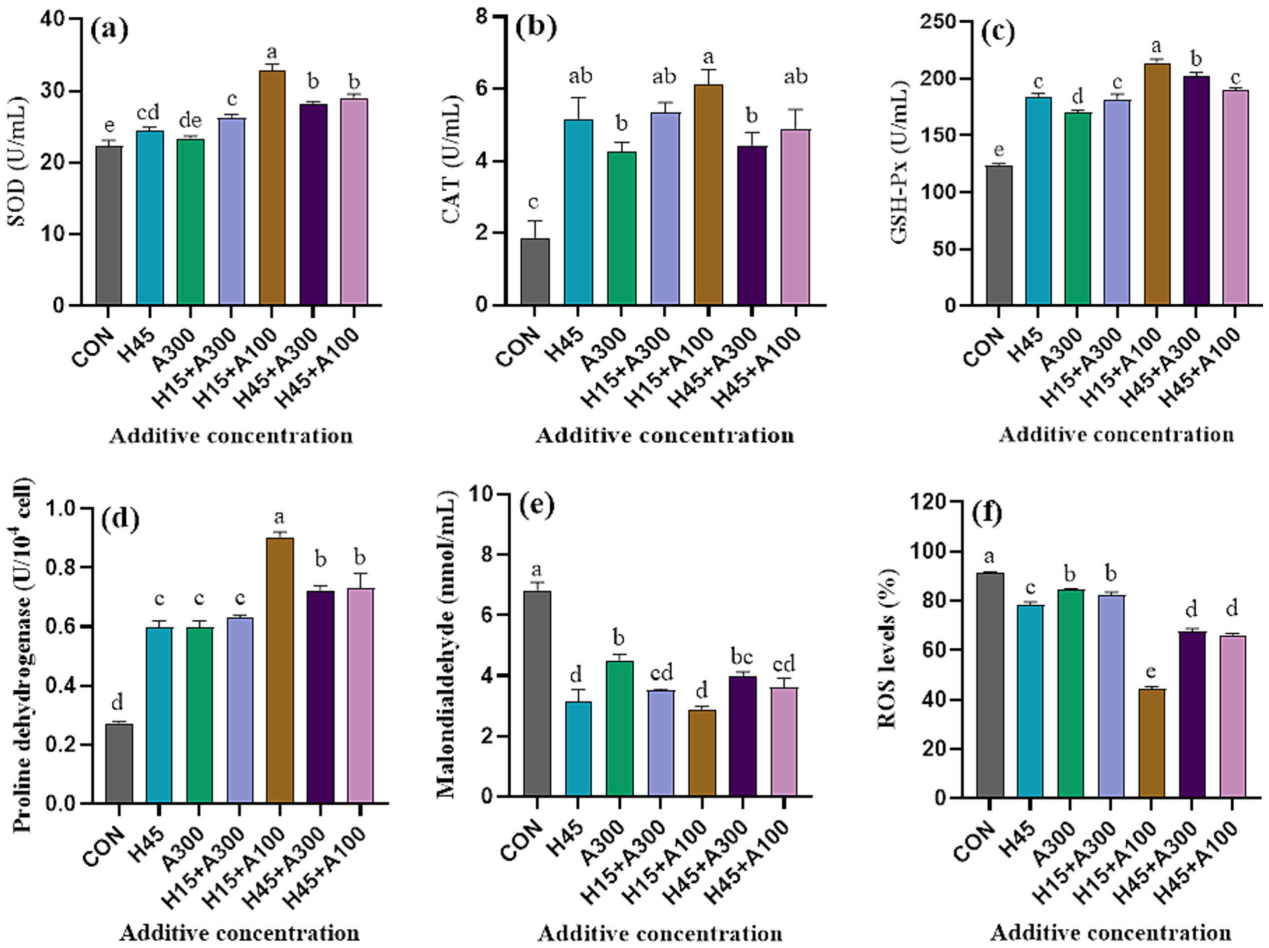
Effects of Hsd and AsA supplementation on the antioxidant parameters of frozen-thawed boar sperm in control and at 10 min after thawing. **(a)** SOD activities, **(b)** CAT activities, **(c)** GSH-Px activities, **(d)** PRODH activities, **(e)** MDA content, **(f)** ROS level.

As shown in Figure 4, SOD activity in Hsd treatment was significantly (*p* < 0.01) greater than that in control group. The addition of Hsd and AsA significantly (*p* < 0.01) increased CAT and GSH-Px activities compared with the control group. The SOD, CAT, and GSH-Px enzyme activities of 15 μM Hsd + 100 μM AsA group were significantly higher than those of the control and other groups. The PRODH activity in all treatments were significantly (*p* < 0.01) higher than that of the control group. Meanwhile, the difference between 15 μM Hsd + 100 μM AsA group and other treatment groups were significant (*p* < 0.01). Compared with the control group, the addition of Hsd and AsA significantly (*p* < 0.01) decreased the levels of MDA and ROS in frozen-thawed sperm. The ROS levels were lowest when 15 μM Hsd + 100 μM AsA were supplemented in the extender.

### 3.4 Effect of supplementation with Hsd and AsA on protein expression of frozen-thawed boar sperm

**Figure 5:**
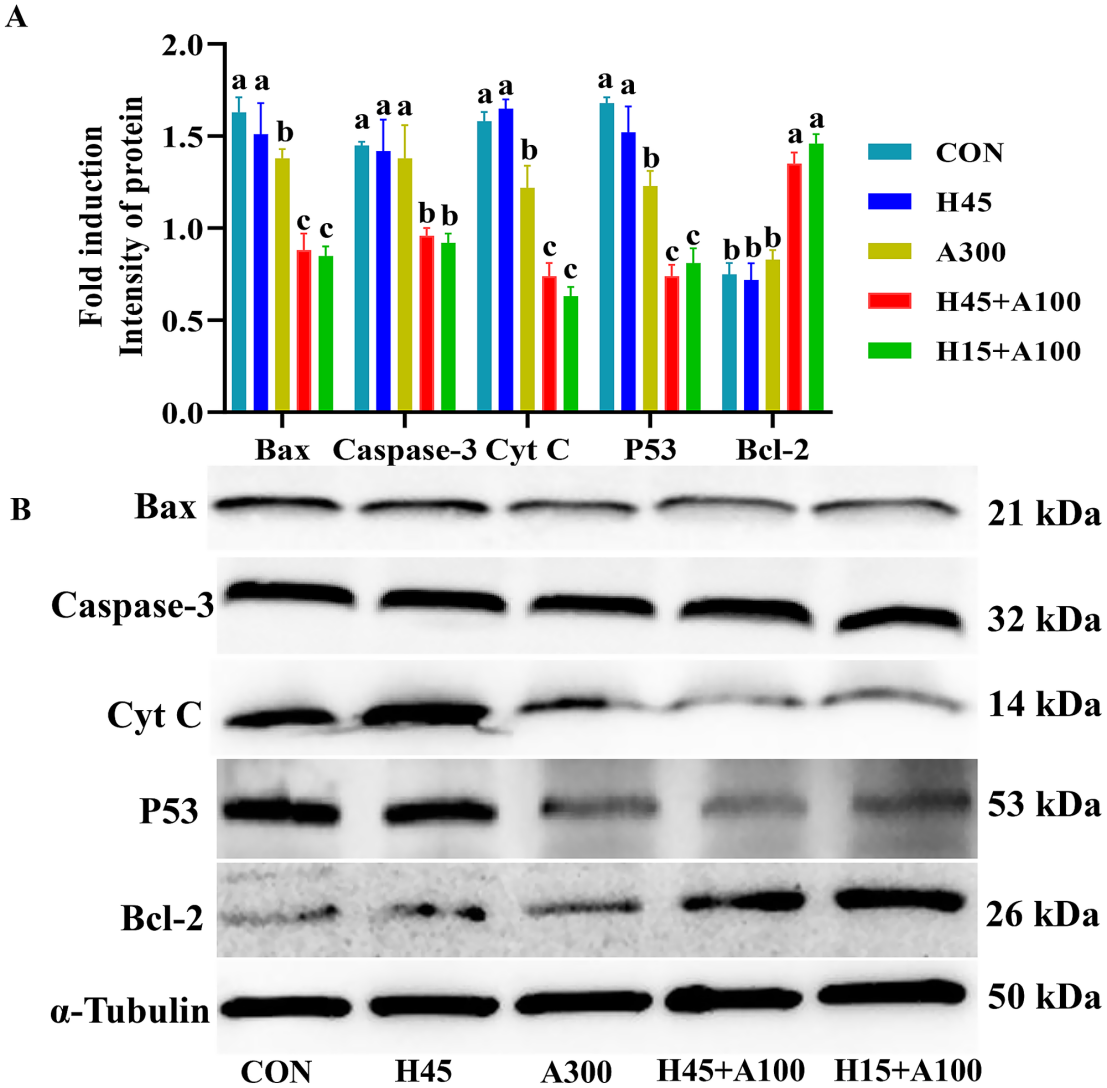
Effects of Hsd and AsA supplementation on part groups of the protein expression of frozen-thawed boar sperm in control and at 10 min after thawing. In image **(A)**, relative quantitative intensity of protein expression of the frozen-thawed boar sperm. In image **(B)**, protein expression of frozen-thawed boar sperm with western blot.

As shown in Figure 5, part groups of the protein expression level of BCL-2 in the frozen-thawed sperm of Hsd combined with AsA group was significantly greater than that of Hsd group alone. Western blotting detected that the expression of apoptotic factor proteins Bax, Caspase-3, P53 and Cytochrome C in Hsd combined with AsA group were significantly decreased (*p* < 0.05, Figure 5B).

## 4 Discussion

China has the largest swine population in the world and accounts for roughly 45% of global pork production and 50% of the consumption of pork (22). African swine fever outbreaks have led to gross losses in the swine industry in China (23). The technology of cryopreservation in boar sperm is widely used to preserve the indigenous breeds, improve the preservation of superior breeds and the transport of genetic material without moving standing animals (2, 24). However, dramatic changes in temperature have induced oxidative damage to boar sperm during cryopreservation. Such damage is caused by the relatively high content of polyunsaturated phospholipids of the membrane and the relatively low antioxidant capacity of boar seminal plasma (6). The effective use of antioxidants during cryopreservation can improve survivability and minimize cryogenic damage. Some researchers have demonstrated the antioxidant function of both Hsd and ascorbic acid (25, 26). In addition, studies have shown that both AsA and other antioxidant combinations significantly improved boar sperm cryotolerance compared to when AsA was separately added to freezing and thawing media (17). This was the first report on the combined effect of Hsd and AsA on boar sperm quality during the freezing-thawing process. In the present study, the combined addition of Hsd and AsA to the boar sperm extender was evaluated. Our results showed that the combination of Hsd with AsA could improve boar sperm motility and velocity compared to when Hsd or AsA was separately added, as compared with the control. A recent study reported that semen motility parameters of Simmental cattle were significantly improved by Hsd supplementation (14). A previous study demonstrated that pig sperm frozen in an extender supplemented with 200 μM ascorbic acid 2-O-α-glucoside significantly increased sperm motility (18). Consistent with the results of the current study, the addition of both 15 μM Hsd and 100 μM of AsA to the freezing extender better protected the sperm from oxidative damage and significantly improved sperm motility.

Boar sperm is sensitive to the damage produced during the freezing-thawing process (27). In addition, because their plasma membranes contain a lot of polyunsaturated fatty acids, sperm cells are more susceptible to oxidative stress (28). It was reported that there was a significant correlation between antioxidant status and quality of frozen-thawed semen (29). The role of Hsd in the quality of frozen-thawed semen was evaluated in recent research, which indicated that Hsd significantly improved semen motility parameters in Simmental bull and reduced oxidative stress status in seminal plasma (14). To our knowledge, few studies have evaluated the effect of Hsd on semen quality after supplementation to an extender medium for frozen-thawed boar sperm. In the present study, treatment with 15 μM Hsd + 100 μM AsA had the greatest antioxidative enzyme activities of SOD, GPx and CAT in the frozen-thawed sperm among the tested groups. They play a crucial role in protecting sperm against the harmful effects of free radicals released due to mitochondrial metabolism. This protection is achieved through the actions of SOD and GSH-Px preventing oxidative attack during freezing. The SOD and GSH-Px are the main antioxidant enzyme defense systems in cells associated with antioxidant capacity. SOD is known to catalyze the decomposition of superoxide into hydrogen peroxide (H_2_O_2_) and oxygen (O_2_), and the increase of SOD activity can protect CAT and GSH-Px from inactivation of O_2_^−^, thus increasing the activity CAT and GSH-Px (29). Adding exogenous antioxidants to freezing media has been suggested as a strategy to counteract the effects of oxidative stress on cryopreserved sperm cells (30). Thus, the addition of Hsd + AsA to freezing and thawing media protects boar sperm against oxidative stress due to their antioxidant properties. Antioxidants possess the ability to combat free radicals by interrupting the reaction chain within the mitochondrial membrane. This interruption ultimately prevents oxidative damage. Consequently, we assume that Hsd and AsA play a significant role in the intracellular mechanism involved in cryopreservation. Cryopreservation is associated with an increase in the generation of reactive oxygen species (ROS) (31). In the reproduction system, high levels of ROS production are more vulnerable to oxidative damage in spite of their greater antioxidant capacity (32). Its cytotoxic effect on membrane phospholipids can lead to the production of malondialdehyde (MDA), which is a last product of unsaturated fatty acid lipid peroxidation (33). The combined addition of Hsd and AsA significantly reduced ROS levels and is more effective than the separate addition of either Hsd or AsA. They act directly or indirectly by minimizing the oxidative damage to lipids, proteins, and nucleic acids caused by ROS (34). Both Hsd and AsA may help increase fertility by reducing oxidative damage to DNA, proteins, and lipids. Membrane lipid peroxidation has been measured using malondialdehyde (MDA) formation. Supplementing freezing media with a Hsd and AsA combination significantly reduced lipid peroxidation. The structurally intact and functionally activated plasma membrane is crucial for the various physiological activities of sperm cells, including metabolism, capacitated acrosome reaction, attachment, and crossing of egg cells. For this reason, Hsd and AsA protect sperm from the detrimental effect of free oxidative radicals and have shown great sperm cryoprotective potential.

Oxidative stress occurs during freezing-thawing damage caused by an imbalance between the generation of ROS and antioxidants (35). Therefore, the intensity and duration of oxidative stress may influence the fate of cells, ranging from adaptive responses to apoptosis or necrosis (36). It can cause DNA damage in boar sperm. If the level of DNA damage exceeds the cells’ repair capabilities, it eventually leads to apoptosis. The decrease in mitochondrial membrane potential indicates early stages of apoptosis. Frozen-thawed sperm exhibited lower membrane integrity and sublethal dysfunction as a result of the cold shock, osmotic stress, and oxidative damage that occurred during cryopreservation. In this study, the supplementation of both Hsd and AsA significantly improved the integrity of the plasma membrane, acrosome, and DNA of frozen-thawed boar sperm compared with the control group. Additionally, the highest improvement was observed when the extender was supplemented with a combination of 15 μM of Hsd + 100 μM of AsA, which was greater when Hsd or AsA was added alone. This is in agreement with a previous study (17), in which combining reduced glutathione and AsA significantly improved acrosome integrity, sperm motility, and the integrity of nucleoprotein structure compared to the one that only contained reduced glutathione or AsA. The addition of a combination of Hsd and AsA to the extender produces a synergistic improvement in post-thawed sperm quality, preventing oxidative damage, lowering lipid peroxidation, DNA damage, and the expression of apoptotic factor proteins. However, this trial suffers from the limitations of the longer time evaluation after thawing.

## 5 Conclusion

In conclusion, the combined addition of Hsd and AsA to the extender enhanced the motility and integrities of the membrane, DNA, and acrosome of boar sperm. The combination of 15 μM Hsd + 100 μM AsA greatly increased mitochondrial and antioxidant functions while decreasing the levels of MDA and ROS and sperm apoptosis after freezing and thawing. The beneficial effect of Hsd combined with AsA on quality of frozen-thawed boar sperm was greater than that of Hsd or AsA alone.

## Data Availability

The original contributions presented in the study are included in the article/supplementary material, further inquiries can be directed to the corresponding author.
